# Sun Exposure and Its Effects on Human Health: Mechanisms through Which Sun Exposure Could Reduce the Risk of Developing Obesity and Cardiometabolic Dysfunction

**DOI:** 10.3390/ijerph13100999

**Published:** 2016-10-11

**Authors:** Naomi Fleury, Sian Geldenhuys, Shelley Gorman

**Affiliations:** Telethon Kids Institute, University of Western Australia, P.O. Box 855, Perth 6872, Australia; Naomi.Fleury@telethonkids.org.au (N.F.); ms.geldenhuys@gmail.com (S.G.)

**Keywords:** obesity, ultraviolet radiation, type-2 diabetes, non-alcoholic fatty liver disease

## Abstract

Obesity is a significant burden on global healthcare due to its high prevalence and associations with chronic health conditions. In our animal studies, ongoing exposure to low dose ultraviolet radiation (UVR, found in sunlight) reduced weight gain and the development of signs of cardiometabolic dysfunction in mice fed a high fat diet. These observations suggest that regular exposure to safe levels of sunlight could be an effective means of reducing the burden of obesity. However, there is limited knowledge around the nature of associations between sun exposure and the development of obesity and cardiometabolic dysfunction, and we do not know if sun exposure (independent of outdoor activity) affects the metabolic processes that determine obesity in humans. In addition, excessive sun exposure has strong associations with a number of negative health consequences such as skin cancer. This means it is very important to “get the balance right” to ensure that we receive benefits without increasing harm. In this review, we detail the evidence around the cardiometabolic protective effects of UVR and suggest mechanistic pathways through which UVR could be beneficial.

## 1. Introduction

**Figure 1:**
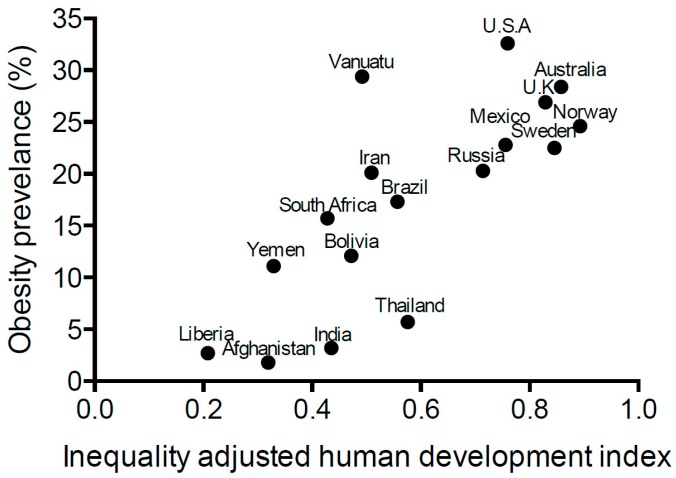
Obesity prevalence. The prevalence of obesity in a selection of countries with varying degrees of development according to the inequality adjusted human development index, which takes into account life expectancy and quality, years and quality of education and the income of the average citizen [4], was calculated using data from the United Nations Development Programme (2015) [5]. Obesity prevalence is accurate as of the year 2014 from data collected by the World Health Organisation [6]. A significant positive linear relationship between human development index and obesity prevalence is observed (*p* = 0.0003, *r* = 0.58; Pearson test).

Obesity is a concerning epidemic worldwide. It is defined as a body mass index (BMI) of over 30 kg/m^2^. In 2014, 266 million men and 375 million women were estimated to be obese worldwide [1]. This figure is expected to continue rise with global increases of ~0.5 kg/m^2^ in both men and women over the past decade [1,2]. If this trend continues there will be virtually no chance of reaching the global obesity target of the World Health Organisation (Target 7), which is to halt the rise in obesity prevalence by 2025. Although obesity tends to have a higher prevalence in developed countries (Figure 1), it increasingly affects the health of citizens in developing countries. Of particular concern is the prevalence of obesity in children and adolescents (under 18 years of age) worldwide, which in 2013 occurred in 24% of boys and 23% of girls [3].

Obesity is associated with a number of chronic health conditions and other co-morbidities including; metabolic syndrome (MetS), type-2 diabetes mellitus, hypertension, dyslipidaemia, cancer, osteoarthritis, stroke, retinopathy, neuropathy, nephropathy, non-alcoholic fatty liver disease (NAFLD) and sleep apnoea. Due to the widespread nature and health harms of obesity there is a need for research into interventions, which are both cost-effective and readily accessible. One such intervention could be the use of safe (low dose) exposure to sunlight or ultraviolet radiation (UVR). In this review, we will give some background as to how obesity is diagnosed and the nature of three comorbidities (metabolic syndrome, type-2 diabetes and NAFLD), the positive and negative impacts of UVR exposure on human health, with a focus on its potential to regulate cardiometabolic dysfunction, and finally discuss (possible) anti-obesogenic mechanisms of UVR.

## 2. Diagnosis of Obesity

Obesity is usually defined as a BMI above 30 kg/m^2^. However, this definition is controversial as it may not accurately represent adiposity [7]. In particular, BMI fails to account for muscle mass. For example, an athlete with high muscle mass but low body fat may be considered obese, whereas an elderly person with high body fat and low muscle mass may be classified as healthy [7]. Waist circumference is also commonly used to classify obesity (>102 cm for men, >88 cm for women) [8] and may be a better predictor (than BMI) of the risk of developing the co-morbidities associated with obesity (including type-2 diabetes) [9]. Skin-fold has also been used to measure obesity. However, this method is inaccurate, difficult to reproduce and hard to measure in people with a BMI > 35. Measuring the waist to hip ratio (>0.55 is obese) is advantageous because it measures central adiposity, a significant risk factor for cardiovascular disease and other co-morbidities of obesity [10]. Ongoing technical advances in imaging, such as dual energy X-ray absorptiometry (DEXA) [11] and air displacement plethysmography [12] may be changing the way adiposity is measured, but are expensive and have other limitations [13]. Obesity is often associated with metabolic disorders, three of which we describe below.

## 3. Metabolic Syndrome

MetS is a cluster of metabolic disturbances, including obesity, insulin resistance, dyslipidaemia and hypertension. In most countries, the prevalence of MetS is between 20% and 30% [14]; however, trying to determine the prevalence of MetS is difficult as there are at least three major definitions in common use [15,16,17], and each has different clinical criteria and cut-off scores to diagnose MetS.

## 4. Type-2 Diabetes

Type-2 diabetes is a common metabolic disorder characterized by chronic hyperglycaemia and is associated with obesity [18]. It is associated with reduced life expectancy owing to a greater risk of heart disease, stroke, peripheral neuropathy, renal disease, blindness and amputation. A predictor of diabetes risk is insulin resistance, a state in which cells (in particular adipocytes, hepatocytes and muscle cells) become resistant to the effects of insulin, causing the pancreas to produce increasing amounts of insulin until it can no longer produce enough to meet the body’s needs [19]. Another predictor is impaired glucose tolerance, which is latent diabetes.

## 5. Non-Alcoholic Fatty Liver Disease (NAFLD)

NAFLD is a “disease state in which lipid accumulates in the liver in the absence of excessive alcohol consumption” [20]. NAFLD is a spectrum of diseases, which range from liver steatosis to severe liver steatohepatitis, which may cause liver cirrhosis [21]. NAFLD is probably the hepatic consequence of MetS and is associated with insulin resistance [21]. As rates of obesity and MetS have increased, so too have rates of NAFLD and it is rapidly becoming the most prevalent liver disease worldwide. The prevalence of NAFLD is difficult to determine, as histopathological examination of a liver biopsy is needed for a definitive diagnosis. Proton magnetic resonance spectroscopy is currently the gold standard and a safe, effective and non-invasive method for determining the accumulation of fat in the liver [22].

## 6. Chronic Low-Grade Inflammation Is Associated with Obesity and Cardiometabolic Disease

Obesity and the chronic disorders of MetS, type-2 diabetes, and NAFLD are associated with chronic and systemic low-grade inflammation [23]. This inflammation may result from adipose tissue hypoxia, which causes the release of pro-inflammatory cytokines [24]. This low-level but ongoing inflammation may contribute towards the development of many co-morbidities of obesity, in particular insulin resistance and NAFLD, and can impair metabolic processes that govern glucose and triglyceride uptake [23]. Hotamisligil and colleagues originally observed that obesity in rodents was associated with elevated circulating tumour necrosis factor (TNF)-α, which may be secreted by white adipose tissue (WAT) [25]. WAT secretes different bioactive molecules called adipokines, including >50 cytokines, chemokines, hormones and other mediators [26]. These include many pro-inflammatory cytokines, including TNFα, interleukin-1 (IL-1), interleukin-6 (IL-6), interferon gamma, monocyte chemotactic protein and high-sensitivity c-reactive-protein (CRP), a sensitive marker of inflammation. These mediators may cause inflammation in pancreatic islets to compromise beta cell function, providing a mechanistic link for obesity and insulin resistance [27]. Indeed, increased circulating levels of CRP, IL-6, and TNF-α occur in individuals with type-2 diabetes and insulin resistance [28]. These adipokines and pro-inflammatory mediators perpetuate inflammation and insulin resistance by activating pathways that involve “nuclear factor kappa-light-chain-enhancer of activated B cells” (NF-κB) [29].

**Figure 2:**
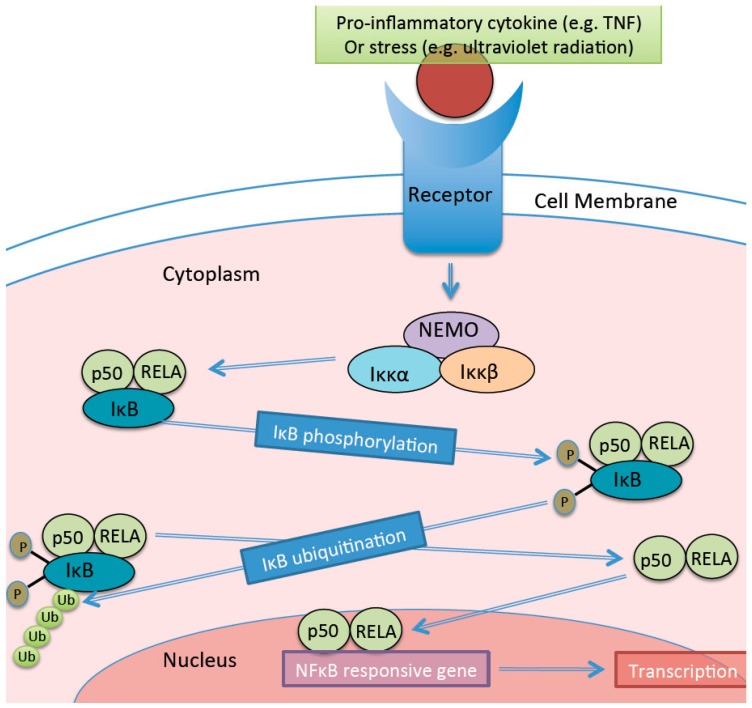
The canonical NF-κB pathway. Pro-inflammatory cytokines or stressors activate a cell receptor (e.g., Toll-like receptors or tumour necrosis factor (TNF)). This cause activation of Iκκβ, which attaches to regulatory proteins like RELA (NF-κB p65 subunit), resulting in the formation of dimers. Iκκβ is activated and transforms into IκB. IκB is then phosphorylated, ubiquitinated, and then degraded. This causes the release of the dimers attached to it (e.g., p50-RELA heterodimer). The dimers are trans-located to the nucleus and bind to NF-κB responsive genes and induce transcription of pro-inflammatory cytokines. This figure was constructed based on information provided in [30,35,36]. NEMO = NF-κB essential modulator.

The NF-κB pathway can be activated in most cells (including hepatocytes and pancreatic beta cells) through canonical (classical) or alternative pathways [30]. Here, we describe the canonical pathway (Figure 2), in which a pro-inflammatory cytokine (such as TNF) or a general stressor (e.g., age) causes activation of Iκκβ (which is part of the NEMO (NF-κB essential modulator)-Iκκα-Iκκβ complex), which then phosphorylates and ubiquitinates the IκB protein, causing its degradation. Dimers previously attached to the IκB (e.g., p50-RELA (NF-κB p65 subunit) heterodimer) are then translocated to the nucleus where they can induce transcription of pro-inflammatory cytokine genes [30]. Obesity is associated with increased NF-κB activation [31,32]. For instance activation of the NF-κB pathway leads to increased transcription of the *CRP* gene [31] causing the release of CRP from human hepatocytes [32], perpetuating injury and oxidative stress, furthering the progression of NAFLD to non-alcoholic steatohepatitis [33,34].

Other pathways that might be responsible for increased tissue inflammation observed during obesity include effects on the gut microbiome. The low-grade inflammation induces changes in the gut microbiome, impairing the barrier function of the intestinal mucosa (a “leaky gut” [37]) and causing an influx of toll-like receptor 4 and 9 agonists (from the gut) into the portal circulation. Sun exposure can also induce the production of pro-inflammatory mediators in skin (reviewed by [38]), but can have anti-inflammatory effects at a systemic level (reviewed by [39]). Below we summarise some of the evidence around the effects of sun exposure and UVR on human health, and discuss the mediators (and immune pathways) induced by sun exposure may could possibly reduce the risk of developing obesity and cardiometabolic dysfunction.

## 7. Sunlight and Ultraviolet Radiation

For thousands of years humans have understood that sunlight has an important and significant impact on human health and disease. Sunlight is composed of three major wavelength bands: visible light (wavelengths of 400–800 nm); UVR (wavelength of 100–400 nm); and, infrared radiation (wavelengths > 800 nm). The wavelengths of UVR are further divided into three main categories: UVA (315–400 nm) and UVB (280–315 nm), which, respectively, comprise 95% and 5% of UV rays that reach the Earth’s surface; and, UVC (100–280 nm), which is prevented from reaching the Earth’s surface by the ozone layer [40]. UVA can penetrate much deeper into the epidermis than UVB with <10% of UVB reaching the basal germinal layer compared to >20% of UVA [41]. The amount of solar UVR that reaches the terrestrial surface at any given place and time is influenced by a variety of factors including; time of day, season, geographic latitude, altitude, cloud cover and surface type [42]. Some of the studies mentioned in this review use these factors (particularly, season and latitude) as indicators (or proxies) for the amount of exposure to UVR.

### 7.1. Negative Consequences of UVR Exposure

The negative health effects of exposure to UVR have been closely examined. These include sunburn and increased risk of skin cancers (melanoma, lip cancer, and keratinocyte cancers) and ocular diseases (cataracts, pterygium, ultraviolet keratitis and conjunctival neoplasm) [43]. Excessive skin exposure can cause skin erythema (reddening of skin), with oedema and tissue inflammation. The total amount of exposure to UVR and excessive exposure resulting in sunburn in childhood and adolescence significantly increases an individual’s risk of developing melanoma (reviewed in [42]). Sub-erythemal (non-burning) doses of UVR also have biological effects. Absorption of UVR by epidermal cells in skin and the eye leads to the production of reactive oxygen and nitrogen species which can damage biomolecules such as membrane lipids and deoxyribonucleic acid (DNA) [44]. UVR (both UVA and UVB wavelengths) directly damages DNA through the formation of pyrimidine dimers. These are the predominant DNA lesions in human skin exposed to UVR [45]. UVA radiation also damages DNA by: (i) inducing reactive oxygen species that causes the oxidation of DNA bases; and (ii) activating the mitogen-activated protein kinase-dependent pathway [45]. UVR can promote tumour growth through immunosuppression [38]. As far as the ability to cause genetic mutation and suppress human immune responses is concerned, at an equal dose, UVB is far more potent (and carcinogenic) than UVA radiation [45]. However, humans are exposed to more UVA than UVB as the relative amounts of UVA (95% of UVR) in sunlight far exceed UVB (5% of UVR). The ability of UVR to cause genomic damage may be an initiating factor in the pathogenesis of skin cancers [46] with a role for reactive oxygen species in UVR-induced eye disease [47]. Exposure to UVA radiation still occurs when the UV index (UVB-weighed) is low (for example in winter in temperature climates, or early morning and late afternoon), or through window glass (which blocks UVB radiation). UVA poses risks to human health because of its capacity to induce pro-inflammatory cytokine production in skin and degrades vitamin D in skin [48]; however, UVA may induce the release of nitric oxide from stores in skin to improve blood pressure (see Section 7.5.2).

### 7.2. Health Benefits of Sun Exposure

Sunlight may be beneficial for the treatment of a number of medical conditions including some cancers and neurodegenerative diseases. Chronic but not acute exposure to UVR was associated with significantly reduced incidence of cancers of the breast, prostate, colon/rectum and non-Hodgkin’s lymphoma [49,50,51,52,53]. Cancer survival is associated with better responses in patients that have higher cumulative sun exposure or who are diagnosed in summer/autumn, which was attributed to the activity of increased circulating 25-hydroxyvitamin D [54,55,56]. Sun exposure is inversely associated with some (but not all) infections, including tuberculosis [57] and acute respiratory tract infections [58]. As detailed below, sun exposure suppresses immunity and may prevent the development and limit the severity of immune-driven diseases.

### 7.3. Autoimmune and Allergic Disease

Increased sun or UVR exposure is associated with reduced development and/or severity of immune-driven diseases including autoimmune diseases such as arthritis [59], inflammatory bowel disease [60,61], multiple sclerosis [62,63,64,65,66,67,68,69], and allergic conditions such as asthma [39,70] and anaphylaxis [71,72]. The evidence around the protective effects of sun exposure in affecting multiple sclerosis is particularly strong, where increased sun exposure [62,63,66,68,73] (and serum 25(OH)D [67,74]) was associated with reduced risk of multiple sclerosis, particularly in childhood and adolescence [64,65,69] There are many pathways by which exposure to UVR suppresses immunity, through multiple mediators and cellular networks, locally and systemically [39]. Some of these mediators and immune network effects may also be beneficial for the control of metabolic dysfunction.

### 7.4. UVR Modulation of Obesity and Related Metabolic Disorders

Below we present findings that may suggest that ongoing exposure to sunlight or UVR radiation may modulate the development of obesity and cardiometabolic dysfunction.

#### 7.4.1. Animal Studies

Animal investigations suggest a protective effect of UVR in reducing weight gain and metabolic dysfunction. We observed that chronic exposure to UVR suppressed weight gain and the development of glucose intolerance, insulin resistance and signs of NAFLD in C57BL/6J male mice fed a high fat diet [75]. UVR was effective at low sub-oedemal (non-burning) doses (1 kJ/m^2^) given twice a week, and at higher oedemal doses (4 kJ/m^2^) administered once a fortnight. In another study, Lewis rats were fed a choline-deficient iron-supplemented L-amino acid-defined diet to induced non-alcoholic steatohepatitis [76]. Ongoing artificial sunlight (UVR) “ameliorated hepatocyte apoptosis, inflammation, fibrosis, and insulin/leptin resistance” when administered for 12 h a day for 6–12 weeks to rats compared to untreated controls, but did not affect weight gain [76]. Key differences between our studies [75] and these [76] include the dose of UVR administered (not reported by [76]) and the animal models, in which obesity was not induced specifically in Lewis rats [76].

#### 7.4.2. Human Studies

We have recently reviewed in detail the evidence from a limited number of human observation studies and clinical trials, which assess the potential for exposure to sunlight or UVR to affect the development of obesity and metabolic dysfunction (invited review, [77]). Below, we detail some of these findings, concentrating on a few key studies published to date.

##### Human Observational Studies

Using latitude and altitude as proxies for sun exposure, some studies suggest a protective effect of decreasing latitude (i.e., living closer to the equator [78]) or increased altitude [79], but not all report a protective effect [80,81]. In other studies, seasonal increases in adiposity, BMI, abdominal obesity and HbA1c levels have been observed in winter [82,83], although there is uncertainty around seasonal effects on insulin secretion and sensitivity [84,85]. Research in pregnant women suggests that increased sun exposure around the time of delivery was associated with a reduced risk of gestational hypertension [86]. Perhaps some of the strongest epidemiological evidence for a protective effect of sun or UVR exposure comes from the MISS cohort (*n* = 24,098) of Swedish women, in which sun-seeking behaviours or the use of sunbeds was associated with a reduced risk of type-2 diabetes [87], thromboembolic events [88] and all-cause mortality [89], independent of physical activity. An issue with these studies was the use of a self-report survey around sun or UVR exposure, which could have been affected by recall bias. Further research using direct measures of sun exposure (through the use of dosimeters), will help define the epidemiological associations between sun exposure, obesity and cardiometabolic dysfunction.

##### Human Experimental Studies

There are very few published studies in which the effects of sun exposure or UVR on obesity and metabolic dysfunction have been directly tested in humans. In a small study performed over 12 months, the incidence of MetS was reduced after a 12-month intervention given to 69 non-diabetic overweight adults from Saudi Arabia, who were advised to regularly expose themselves to sunlight and to eat more vitamin D-rich foods [90]. This study suggests that there may be a protective effect of sun exposure (and eating vitamin D-rich foods) in reducing MetS incidence; however, there was no placebo control arm. Other researchers have noted no significant improvements in body mass or fat in overweight psoriasis patients receiving narrow-band UVB therapy; but, again, there was no control group, making it difficult to accurately determine the effects of UVR [91]. A limited number of small clinical trials have demonstrated protective effects of controlled UVR (UVA or UVB) on hypertension [92,93,94]. However, more research is clearly needed in people to determine the relationships and direct effects sun or UVR exposure and the risk of developing obesity, type-2 diabetes, MetS and NAFLD.

### 7.5. Pathways through Which UVR Could Suppress Obesity and Signs of Cardiometabolic Dysfunction

Many of the beneficial effects of UVR are presumed to be through vitamin D. Exposure to UVR induces many other mediators [39], which may be important for optimal musculoskeletal health. Below we discuss the potential for UVR-induced vitamin D, nitric oxide, α-melanocyte-stimulating hormone (through the pro-opiomelanocortin pathway) and heme-oxygenase to affect body weight and metabolic function. We also pose a role for UVR in modulating adipose inflammation through potential effects on regulatory T cells.

#### 7.5.1. UVR-Induced Vitamin D

**Figure 3:**
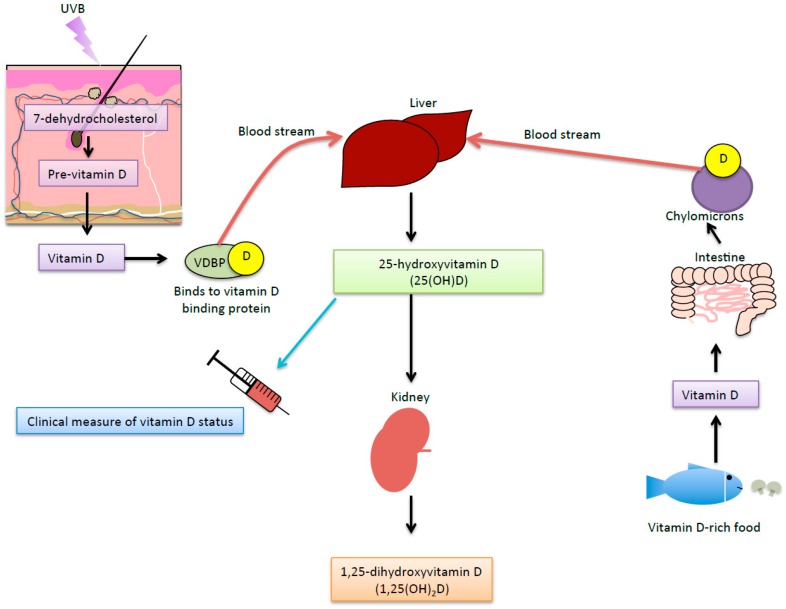
Vitamin D synthesis. When skin is exposed to UVB radiation, 7-dehydrocholesterol (in the skin) transforms into pre-vitamin D that isomerises with body heat into vitamin D. Free vitamin D binds to the vitamin D binding protein (VDBP), which is transported through the blood stream into the liver and is hydroxylated to form 25-hydroxyvitamin D (25(OH)D). This form of vitamin D is used as the clinical measure of vitamin D status. 25(OH)D is then transported to the kidney (and other tissues) where it is hydroxylated to form the biologically-active product 1,25-dihydroxyvitamin D (1,25(OH)_2_D). Additional vitamin D can be obtained from vitamin D-rich food sources (such as fish and mushrooms) and supplements. Vitamin D is absorbed through the intestines into the bloodstream in chylomicrons. Figure 3 was constructed based on information provided in [42,95].

Vitamin D is a fat-soluble hormone that is vital for maintaining human health, as it is needed for homeostasis of the plasma levels of calcium and phosphorus. When human skin is exposed to UVB radiation, vitamin D_3_ is produced from 7-dehydroxycholesterol via pre-vitamin D_3_ (Figure 3). Modifiers of UV-induced vitamin D synthesis include the UV index (latitude, time of year or day) and personal factors (e.g., skin colour, clothing, and genetics). Vitamin D can also be obtained through dietary intake of vitamin D-rich foods or supplements, although most people acquire most of their vitamin D through sun exposure (Figure 3).

Vitamin D is required for optimal bone health with deficiency associated with a range of bone diseases such as rickets, osteopenia, osteoporosis and oesteomalacia [96]. Vitamin D deficiency is defined as 25-hydroxyvitamin D (25(OH)D) levels less then 50 nmol/L. Lower circulating 25(OH)D levels are associated with obesity [97], NAFLD [98,99], insulin resistance [100], MetS [101], type-2 diabetes [102] and cardiovascular disease [103]. There are a number of possible reasons for reduced serum 25(OH)D levels in obese people, including: sun-avoiding behaviours [104], reduced dietary intake of vitamin D [105]; changes in vitamin D metabolism [105]; and, reduced bioavailability of vitamin D through storage in adipose tissue [106]. There are also plausible pathways through which metabolism in fat cells can be modulated by vitamin D. Adipocytes express vitamin D receptors (like many other cells) and are therefore responsive to the active vitamin D metabolite, 1,25-dihydroxvitamin D (1,25(OH)_2_D) in vitro [107]. However, meta-analyses of clinical trials do not support that there are benefits of supplementation with vitamin D for weight loss, coronary artery disease or hypertension, or reducing signs of cardiometabolic risk such as glucose tolerance, insulin sensitivity or circulating lipids [100]. It may be that reduced 25(OH)D levels are a marker of ill health [100]. Serum 25(OH)D are reduced in patients who have experienced a severe inflammatory insult (e.g., post-knee transplant surgery or during an episode of pancreatitis) [108,109], suggesting that inflammation reduces 25(OH)D. Another hypothesis is that chronic conditions may be associated with poor lifestyle choices such as smoking and being sedentary, which may in turn cause vitamin D deficiency [100]. Alternatively, 25(OH)D levels could be a proxy for sun exposure, and other UVR-induced mediators (e.g., nitric oxide) may be responsible for the positive health outcomes associated with increased circulating 25(OH)D.

#### 7.5.2. UVR-Induced Nitric Oxide

**Figure 4:**
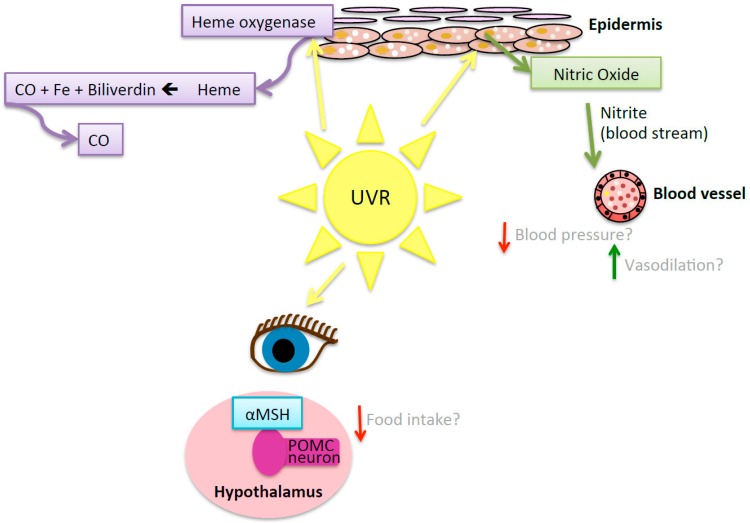
Mediators other than vitamin D are produced or released by exposure to UVR and may play a role in reducing weight gain and signs of cardiometabolic dysfunction. Exposure to ultraviolet radiation (UVR) results in the production of heme oxygenase, which causes the breakdown of heme, catalysing the production of carbon monoxide (CO), iron (Fe) and biliverdin; however, the role of this pathway on the development of obesity and cardiometabolic dysfunction is still to be defined. Nitric oxide stores in skin are released into the blood stream as nitrite potentially reducing blood pressure and increasing vasodilation. When skin and the eye are exposed to UVR there is a release of α-melanocyte-stimulating hormone (MSH), which activates pro-opiomelanocortin (POMC)-responsive neurons in the arcuate nucleus of the hypothalamus, and hypothetically could reduce appetite and food intake.

Skin exposure to UVR triggers the release of nitric oxide from dermal storage sites into the blood stream [110], which can be measured by increases in serum nitrite [111,112] (Figure 4). Anti-hypertensive and vasodilatative effects are induced by treatment with nitric oxide and compounds that increase local levels of nitric oxide-related metabolites, such as nitrite or nitrate [110,113]. Whole-body irradiation of healthy adult volunteers to UVA radiation reduced blood pressure in healthy young adult males, which was sustained for 30 min. These effects were associated with an increase in circulating nitrite [111]. The effects of UVA radiation were independent of nitric oxide synthase, suggesting a role for the release of preformed nitric oxide stores from cutaneous tissue through the alternate nitrate-nitrite nitric oxide pathway. Nitric oxide induced by eye exposure to UVR can suppress immunity in a systemic fashion [114,115].

Our studies suggest that UVR-induced nitric oxide can have benefits for the control of the cardiometabolic dysfunction associated with obesity. To demonstrate a role for UVR-induced nitric oxide, a nitric oxide scavenger (2-(4-Carboxyphenyl)-4,4,5,5-tetramethylimidazoline-1-oxyl-3-oxide potassium salt, cPTIO) was applied to irradiated skin immediately following exposure to UVB radiation (1 kJ/m^2^) also administered to mice fed a high fat diet [75]. As an alternative to UVR, some mice received a topical treatment twice a week with a nitric oxide donor (*S*-nitroso-*N*-acetylpenicillamine, SNAP). The UVR or SNAP treatments (alone) increased skin nitric oxide levels 5 min after skin exposure, while cPTIO treatment post-UVR reduced skin nitric oxide levels. In mice fed a high fat diet, the SNAP treatment reduced mouse weights and the development of insulin resistance, while topical cPTIO reversed some of the positive effects of UVR, specifically, fasting glucose levels and hepatic steatosis; suggesting that some of the benefits of UVR may be dependent on skin release of nitric oxide [75]. Results from other rodent studies suggest that dietary nitrate causes browning of WAT and increased expression of thermogenesis (heat production)-related genes in brown adipose tissue [116], providing a mode of action through which dietary nitrate has anti-obesogenic effects [117]. Other studies support the notion that increasing the bioavailability of nitric oxide may have benefits for obesity, with reported benefits of nitrate-rich supplements in reducing circulating triglyceride levels [118]. However, the relationships between obesity and nitric oxide are complex, where bioavailability of nitric oxide may be reduced in obese individuals compared to healthy age- and gender-matched counterparts [119], and excessive expression of nitric oxide (known as nitrosative stress) is associated with tissue inflammation in conditions like NAFLD [120].

#### 7.5.3. Pro-Opiomelanocortin Pathway

Pro-opiomelanocortin (POMC) is a polypeptide secreted by the pituitary gland, skin cells and neurons, which undergoes cleavage into several peptides, including α-melanocyte stimulating hormone (α-MSH), β-endorphin and adrenocorticotrophin [114]. Skin or eye exposure of mice to UVA radiation increased serum levels of α-MSH (Figure 4). There is a surge in α-MSH and melanocortin receptor-4 expression in the arcuate nucleus of the hypothalamus following the exposure of the skin of mice to UVR [121]. α-MSH may help prevent obesity by suppressing appetite and enhancing catabolic signals to promote energy consumption through melanocortin receptors 3 and 4 [122,123]. Exposure to UVR is also followed by an increase in other products of POMC such as β-endorphin [115,121,124,125] and adrenocorticotrophin [124] in the skin, serum and hypothalamus. While α-MSH suppresses appetite, β-endorphin has the opposing effect [126]. The effects of exposure to UVR on appetite and food intake are not known.

#### 7.5.4. Heme-Oxygenase

Heme-oxygenase is an enzyme released in response to cellular stress including that induced after exposure to UVR [127] (Figure 4). Heme-oxygenase catalyses heme into iron, carbon monoxide and biliverdin [110,128]. Carbon monoxide inhibits the production of pro-inflammatory cytokines including TNFα and IL-1β, exerting an anti-inflammatory effect [129]. Chronic treatment of C57BL/6 mice with intraperitoneally injected carbon monoxide (CORM-a1) attenuated the development of obesity (no adverse effects were reported) [130]. Additionally, biliverdin can have an antioxidant effect [128] by reducing bilirubin [131,132]. Bilirubin then reacts with oxy-radicals producing more biliverdin and the cycle continues [131,132]. Treatment with bilirubin (intravenous) reduced body weight, blood glucose levels and total cholesterol levels in a diet-induced murine model (C57BL/6) of obesity [133]. The role of the heme oxygenase pathway in mediating the effects of UVR in preventing the development of obesity and metabolic dysfunction in animal models of obesity are yet to be determined.

#### 7.5.5. UVR-Induced Regulatory T Cells

**Figure 5:**
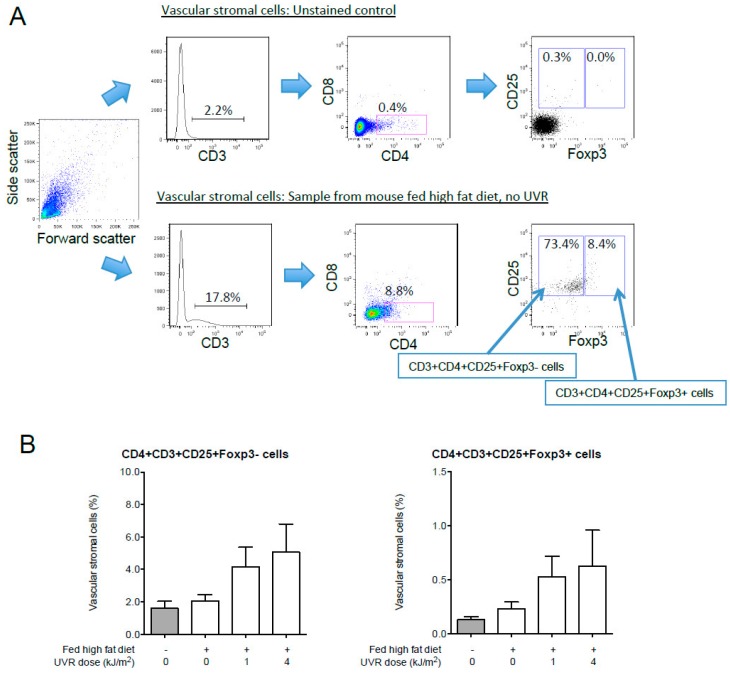
UVR may increase proportions of CD4+ T cells in WAT. Tissue was sampled from C57BL/6J male mice fed a low or high fat diet for 12 weeks. Some mice were exposed to 1 kJ/m^2^ UVR (sub-oedemal dose, two times a week) or 4 kJ/m^2^ UVR (oedemal dose, once a fortnight) for this 12-week period. The mice were from experiments described in [75]. Vascular stromal cells were isolated from gonadal WAT following digestion with collagenase IV (see [139]). Cells (10^6^) were incubated with CD3-FITC, CD25-APC, CD4-PE-Cy5, Foxp3-APC and CD8-APC-Cy7 using previously described methods [139], and then acquired on a LSRII flow cytometer (BD Biosystems). (**A**) The gating strategy, with an example of the forward and side scatter of vascular stromal cells shown in the far left panel, with CD3^+^ and then CD4^+^CD8^−^ cells selected, and cells then divided into CD25^+^Foxp3^−^ and CD25^+^Foxp3^+^ populations. Examples are shown of an unstained control sample, and a sample from a mouse fed the high fat diet and not exposed to UVR for 12 weeks; (**B**) Percentages of CD4^+^CD3^+^CD25^+^Foxp3^−^ and Foxp3^+^ cells, calculated by multiplying the percentages of CD3^+^, CD4^+^CD8^−^ and CD25^+^Foxp3^±^ cells (*n* = 6 mice/treatment, mean + SEM).

UVR exposure has anti-inflammatory systemic effects (reviewed by [39]), promoting the suppressive activity of regulatory T (T_Reg_) cells [134], myeloid cells [135] and other immune cells [136]. Ongoing artificial light therapy reduced hepatic expression of pro-inflammatory genes like TNFα (and potentially NF-κB signalling) in Lewis rats with non-alcoholic steatohepatitis induced by feeding them a choline-deficient and iron-supplemented L-amino acid-defined diet [76], suggesting that UVR might suppress NF-κB and/or other pathways that contribute towards liver inflammation. The capacity of UVR to modulate obesity-induced inflammation in other tissues such as the pancreas and WAT is yet to be determined. T_Reg_ cells in WAT improve insulin sensitivity in an IL-10-dependent fashion [137] and may regulate insulin resistance by modulating the gut microbiome [138]. We have shown that ongoing exposure to UVR impairs the severity of insulin resistance induced in mice fed a high fat diet [75]. Preliminary results from these studies suggest regular exposure to UVR may affect the T cell compartment of WAT. In experiments described in [75], we examined the effects of UVR on CD4^+^ T cell percentages among the vascular stromal cells of WAT of C57BL/6J mice fed a high (or low) fat diet for 12 weeks. The percentages of CD4^+^CD3^+^CD25^+^Foxp3^+^ T_Reg_ cells and CD4^+^CD3^+^CD25^+^Foxp3^−^ T effector (T_Eff_) cells were compared in unexposed mice, mice exposed to 1 kJ/m^2^ UVR (sub-oedemal dose, two times a week) or mice exposed to 4 kJ/m^2^ UVR (oedemal dose, once a fortnight) using flow cytometry (Figure 5A for gating strategy). There was a trend for increased proportions of both CD4^+^CD3^+^CD25^+^Foxp3^+^ T_Reg_ cells and CD4^+^CD3^+^CD25^+^Foxp3^−^ T_Eff_ cells in WAT when collected from mice fed a high fat diet after 12 weeks of chronic exposure to UVR (Figure 5B, *n* = 6 mice/treatment). While further studies with increased numbers of mice per group are required, these observations suggest that the effects of chronic UVR in WAT may not just limited to T_Reg_ cells, but also other populations of T cells, whose role in modulating tissue inflammation and insulin resistance in WAT are yet to be determined.

## 8. Conclusions

Recent estimates suggest that there are ~650 million people worldwide living with obesity [1], which is associated with a number of life-threatening and life-limiting co-morbidities including type-2 diabetes, cardiovascular disease, and NAFLD. Obesity is a significant health and fiscal burden around the world. Animal studies suggest that chronic exposure to UVR could prevent obesity and cardiometabolic dysfunction [75]. However, more research is required to determine the efficacy of safe sun exposure or controlled exposure to UVR in reducing signs of adiposity and cardiometabolic dysfunction in people, particularly in the form of clinical trials. If shown to be efficacious, it is unlikely that any benefits of sun exposure could be obtained through dietary vitamin D supplementation alone. UVR-induced nitric oxide may reduce some aspects of metabolic dysfunction induced by a high fat diet [75] in mice, and we anticipate an important role for other mediators like α-MSH and heme-oxygenase.
